# Thermal Imaging and Infrared Thermometry to Assess Post-wash Cooling: Method Development and Standardization

**DOI:** 10.7759/cureus.87881

**Published:** 2025-07-14

**Authors:** Maheshvari N Patel, Nayan Patel, Apeksha Merja

**Affiliations:** 1 Clinical Research, NovoBliss Research Private Limited, Ahmedabad, IND; 2 Pharmacology, Swaminarayan University, Ahmedabad, IND; 3 Dermatology, NovoBliss Research Private Limited, Ahmedabad, IND

**Keywords:** cooling effect, infrared thermography, skin temperature, thermal camera, thermal imaging

## Abstract

The cooling sensation imparted by topical rinse-off formulations, such as facial cleansers, represents a critical sensorial attribute influencing consumer perception and product acceptability. Despite its subjective nature, this effect can be objectively quantified using non-invasive tools such as infrared (IR) thermography and non-contact infrared thermometry.

The aim was to develop and internally standardize a reproducible methodology for assessing the immediate cooling effect of rinse-off facial products using IR thermal imaging and non-contact infrared temperature measurement.

An exploratory, non-randomized, open-label, crossover evaluation was conducted on 20 healthy male volunteers (aged 22-40 years). Each participant underwent facial skin temperature assessments at three predefined time points: baseline (resting state), post-exercise (to elevate skin temperature physiologically), and immediately after facial cleansing. The test arm involved application of a commercial rinse-off face wash, while the control arm followed cleansing with water only. Facial skin surface temperature was recorded using both a high-resolution IR thermographic camera and a point-based non-contact infrared thermometer.

A statistically significant reduction in mean facial skin temperature was observed post-exercise in both the test and control arms, suggesting localized evaporative cooling. Following product application, the test arm demonstrated a more pronounced decrease in temperature compared to the control. Notably, IR thermography captured broader, region-specific thermal variations and greater magnitude of change than non-contact thermometry, which registered only minor point-specific differences.

The findings support the feasibility and sensitivity of infrared thermography as a robust, objective technique for evaluating the immediate cooling effects of rinse-off cosmetic products. This internally standardized method may be reliably applied in future clinical research to substantiate thermoregulatory or sensorial efficacy claims associated with topical formulations. Specifically, its application may aid in the comparative assessment of novel cooling agents, guide formulation optimization, and support regulatory submissions or marketing claims related to cooling efficacy in both dermatological and cosmetic product development.

## Introduction

The cooling effect of topical facial products, such as face washes, is a key sensory attribute that enhances user satisfaction and product perception. This sensation, often experienced immediately after use, is influenced by formulation components such as menthol derivatives, alcohols, or volatile oils, as well as physical factors like product viscosity and skin interaction [1,2].

Internal prototype evaluation is an established approach in clinical and cosmetic research, providing a structured means to assess the feasibility of study procedures, validate operational workflows, and identify potential challenges before actual subject enrolment [3]. In dermatological settings, particularly when measuring subjective responses like cooling, objective tools such as infrared (IR) thermographic imaging and non-contact thermometers have proven valuable. IR thermographic imaging has emerged as a reliable, non-contact method to monitor surface temperature changes in response to topical product application, providing real-time, visual thermal data. These instruments enable quantification of surface temperature changes with high sensitivity and reproducibility [1,4].

IR thermographic imaging has emerged as a reliable, non-contact tool in dermatological and cosmetic research for objectively assessing surface temperature changes, particularly in studies evaluating cooling sensations from topical products. While prior studies have demonstrated its utility, many lacked standardized protocols and relied on limited or qualitative assessments, reducing reproducibility. The present study addresses these gaps by employing a systematically validated, internally standardized IR thermography method to quantitatively evaluate the immediate cooling effect of a rinse-off cosmetic product, thereby enhancing methodological consistency and applicability in future clinical research.

Physical exercise is known to elevate skin surface temperature through mechanisms such as increased peripheral blood flow and thermoregulatory vasodilation. Incorporating exercise prior to product application allows for a more dynamic temperature profile, thereby improving the ability to detect subtle cooling responses [5]. This contextual insight informed the structure of our internal exploratory method standardization.

Previous studies have shown that exercise-induced elevation in skin temperature can enhance the sensitivity of thermal assessments in cosmetic testing. Moderate physical activity increases peripheral blood flow and raises skin surface temperature, creating a consistent baseline from which the cooling effects of topical products can be more accurately detected. This approach has been used in earlier research to amplify thermal contrast and improve the reliability of IR thermographic measurements. Incorporating exercise as a preconditioning step thus provides a practical and reproducible method to strengthen the sensitivity of cooling efficacy evaluations in cosmetic studies [6].

This internal method development and standardization exercise aimed to establish a reliable and objective procedure for assessing the cooling effect of rinse-off products. The primary objective was to evaluate changes in facial skin surface temperature using two complementary non-invasive tools, an IR thermal imaging camera and an IR thermometer, across three defined time points: baseline (pre-exercise), post-exercise, and post-cleansing. This approach was designed to ensure methodological consistency, reproducibility, and applicability in future controlled clinical trials assessing thermoregulation and perceived cooling effects of topical rinse-off products.

## Technical report

Study design

An exploratory, open-label, two-arm, non-randomized, crossover study was conducted at a single centre to internally develop and standardize a methodology for assessing the immediate cooling effect of topical rinse-off formulations.

Study details

This internal method development and standardization exercise was conducted by the Clinical Trials Department of NovoBliss Research Private Limited, Ahmedabad, on February 25 and 26, 2025. The objective was to develop and standardize an approach for assessing the immediate cooling effect of rinse-off cosmetic products using non-invasive thermal assessment tools. A total of 20 healthy male volunteers aged between 20 and 40 years participated in this exploratory evaluation. The methodology was implemented over two consecutive days: procedures involving the control arm (water wash) were conducted on February 25, 2025, and the test arm (rinse-off formulation) was evaluated on February 26, 2025. All assessments were carried out in a temperature-controlled environment to reduce external variability. Ambient conditions were monitored and documented during both sessions to ensure consistency. On February 25, 2025, the mean room temperature was recorded at 23.6 °C with a relative humidity of 38%, whereas on February 26, 2025, the corresponding values were 24.3 °C and 39%, respectively. These closely matched environmental conditions supported the reliability and comparability of thermal measurements obtained across study arms.

Ethical consideration

This preliminary internal evaluation was conducted on 20 healthy volunteers with the primary objective of method standardization for future clinical investigations. The study was limited to assessing the immediate cooling effect of the rinse-off products using non-invasive IR thermography techniques. The activity was categorized as a procedural quality control initiative rather than biomedical research, as it did not involve systematic investigation aimed at producing generalizable knowledge, nor did it evaluate therapeutic efficacy or cosmetic outcomes in a clinical population. Consequently, in alignment with international research ethics guidelines such as those outlined in the Council for International Organizations of Medical Sciences guideline, this internal method development activity did not require prior approval from an institutional ethics committee [7].

Inclusion and exclusion criteria

The study included healthy male volunteers aged between 20 and 40 years who were willing to comply with the study procedures and provided written informed consent. Participants were required to have no history of facial dermatological conditions that could interfere with thermal assessments and had to be capable of performing light physical activity, such as step-ups and push-ups. Individuals were excluded if they had a fever or systemic illness at the time of evaluation, had undergone recent facial treatments or procedures within the past seven days, or had known allergies or hypersensitivity to any components of the test product, including menthol, kaolin, or green tea extract. Additional exclusion criteria included the use of topical medications or cosmetics on the face within 24 hours prior to the study and participation in any other clinical evaluations within the preceding two weeks.

Temperature assessments

In this evaluation, skin surface temperature was assessed using two non-contact IR devices: a thermal imaging camera (Metravi Pro TI-10P, Software V2.91; Metravi Instruments, Kolkata, West Bengal, India) and an IR thermometer gun (Model NI/406; Naulakha Industries, Delhi, India). IR thermal imaging is a non-invasive technique that detects the naturally emitted IR radiation from the skin, corresponding to its surface temperature. The thermal camera captures this radiation via an IR sensor and generates thermographic images, wherein temperature variations are represented as color gradients-warmer regions typically appearing in red or white, and cooler areas in blue or black. Prior to use, the thermal camera was calibrated using the manufacturer's standard blackbody reference, and the device’s accuracy (±0.5 °C) was verified using internal control procedures. To ensure consistency, all imaging was performed by the same trained operator, and intra-operator reliability was maintained by standardizing the distance (87 cm), angle (0°), and ambient conditions across all captures.

The IR thermometer gun (Model NI/406) operates on the principle of blackbody radiation. An optical lens focuses IR radiation from the skin onto a thermopile detector, which converts the radiation into an electrical signal that is subsequently translated into a temperature value. The thermometer, with an accuracy of ±0.2 °C, was checked against a standard reference body to confirm calibration. Point-specific temperature measurements were consistently recorded from the central forehead region to minimize variability. To reduce inter-operator variability, a single trained evaluator performed all temperature readings using a standardized measurement protocol. This dual-modality approach, combined with appropriate calibration and operator control, enabled a comprehensive and reliable assessment of the immediate cooling effect of the facial cleanser under investigation. 

Experimental procedure

To simulate physiological variations in skin temperature and create a controlled condition for evaluating the cooling effect of a rinse-off facial product, participants underwent a brief standardized exercise regimen. A standardized 10-minute physical activity regimen was implemented prior to product application to induce a consistent elevation in facial skin temperature through thermoregulatory activation. This regimen included 5 minutes of step-up exercises followed by 5 minutes of push-ups. The combination of aerobic and resistance movements was selected based on internal pilot observations to ensure uniform engagement of both upper and lower body muscle groups, thereby promoting peripheral circulation without causing participant fatigue. The duration and sequence were uniformly applied to all participants to minimize inter-subject variability and establish a reproducible physiological baseline for subsequent thermal assessments. This approach was employed to ensure consistency in temperature assessment before and after product application.

Study design and method standardization

Skin surface temperature measurements were taken at three predefined time points: (1) baseline (pre-exercise), (2) immediately post-exercise, and (3) immediately after face wash application. At each time point, facial skin temperature was measured independently using both an IR thermographic camera (Metravi Pro TI-10P) and a non-contact IR thermometer (Model NI/406).

**Figure 1:**
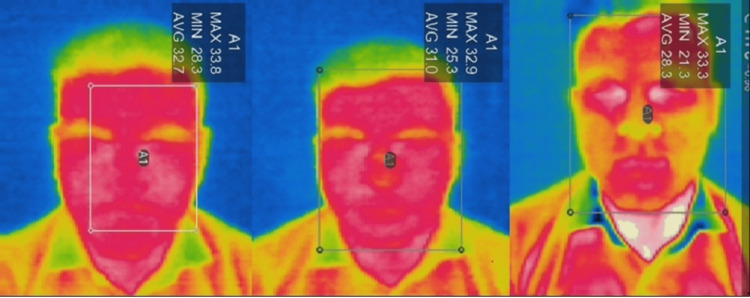
Infrared thermographic assessment in treatment group

**Figure 2:**
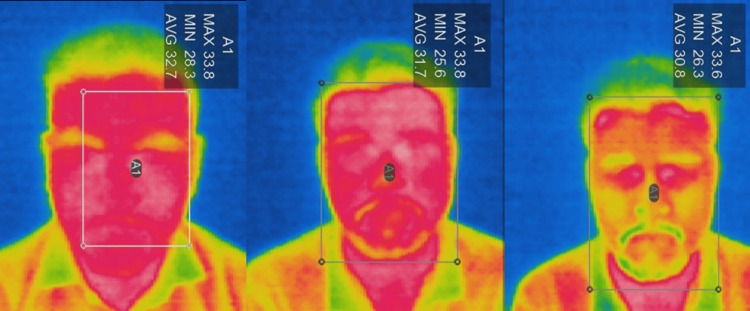
Infrared thermographic assessment in control group

IR thermographic camera and IR thermometer readings were first captured under resting conditions at baseline. Participants then underwent a 10-minute physical activity session, and immediately following exercise, temperature measurements were again recorded. Finally, participants washed their face with the test face wash and gently patted dry using a muslin cloth. Post washing, temperature measurements were captured using both modalities to assess the immediate cooling effect. In the control group, volunteers washed their faces with water only (Figures 1, 2)

All assessments were performed in a temperature-controlled room to minimize environmental variability, and image capture was standardized in terms of IR thermographic camera distance (87 cm away from the object), angle (0°), and lighting. The use of both an IR thermographic camera and an IR thermometer (Model NI/406) enabled objective and reproducible evaluation of temperature changes over time.

Test product

The marketed test face wash (rinse-off product) used in this method development and standardization was formulated with menthol, kaolin, and *Camellia sinensis* (green tea) leaf extract, known for their sensorial and soothing properties. The inclusion of menthol is particularly relevant due to its ability to activate thermoreceptors and elicit a cooling sensation on the skin. For product application, volunteers were instructed to wet the facial area with water, dispense an approximately coin-sized amount of the test product, gently massage it over the entire face, and rinse thoroughly with water. The skin was then gently patted dry using a clean muslin cloth to avoid friction-induced thermal variation prior to instrumental assessment.

Sample size determination 

A total of 20 healthy male participants were enrolled through a convenience sampling method. This approach was appropriate considering the objective of the study, which was to standardize and validate thermal assessment methodologies rather than evaluating therapeutic efficacy. The chosen sample size was sufficient for methodological validation, enabling consistent and reliable comparison across predefined time points. Notably, all 20 participants completed the evaluation and were included in the final data analysis.

Statistical analysis 

Descriptive statistics (N, mean, standard deviation, median, minimum, and maximum) were used to describe continuous variables. Comparisons within and between groups were performed using a paired t-test and an independent t-test, respectively. 

Results

Change in Skin Surface Temperature

Facial skin surface temperature was assessed in 20 healthy male participants using both a non-contact IR thermometer and an IR thermographic camera at baseline, post-exercise, and immediately after face wash application.

Facial skin surface temperature was assessed using a non-contact IR thermometer at three distinct time points: baseline, post-exercise, and post-cleansing. In the test arm (rinse-off product), the baseline mean temperature was recorded as 36.83 ± 0.20 °C. Following the exercise-induced thermogenic stimulus, a statistically significant reduction in mean temperature to 36.44 ± 0.39 °C was observed (change from baseline {CFB}: -0.39 °C; p < 0.0001). Subsequent facial cleansing with the rinse-off product led to a further decrease in mean temperature to 36.45 ± 0.64 °C (CFB: -0.31 °C; p < 0.01), indicating a perceivable immediate cooling effect. In the control arm (water-only cleansing), the baseline mean temperature was 36.56 ± 0.42 °C. Post-exercise, the mean temperature showed a non-significant change to 36.46 ± 0.39 °C (CFB: -0.11 °C; p > 0.05). After cleansing with water alone, the temperature decreased to 36.24 ± 0.40 °C (CFB: -0.32 °C; p < 0.05), reflecting a slight cooling effect attributable to water rinse. The descriptive statistics of the average thermal change are illustrated in Figure 1.

**Table 1:** Average thermal change (descriptive statistics) "Average" refers to the mean facial skin surface temperature (°C) assessed using the infrared thermal camera CFB: change from baseline

Statistics	Before	After exercise	Individual CFB	Individual %CFB	After facewash	Individual CFB	Individual %CFB
N	20	20	20	20	20	20	20
Mean	32.22	31.60	-0.62	-1.92	29.85	-2.37	-7.33
SD	0.85	0.83	0.28	0.87	1.10	1.03	3.15
Median	32.35	31.80	-0.60	-1.84	29.80	-2.40	-7.29
Min	31.00	30.40	-1.50	-4.62	28.10	-4.80	-14.50
Max	33.60	32.80	-0.20	-0.64	31.30	-0.30	-0.96
p-value		0.0000	-	-	0.0000	-	-
Confidence interval		(-0.75, -0.49)	-	-	(-2.85, -1.89)	-	-
p-value vs control		-	0.8834	-	-	0.0000	-
Confidence interval		-	(-0.22, 0.19)	-	-	(-1.97, -0.91)	-
X time		-	1.02	-	-	2.55	-
X%		-	-2.48	-	-	-154.84	-

**Figure 3:**
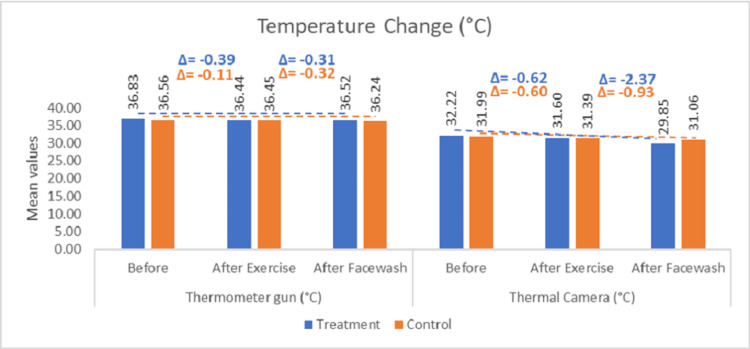
Change in temperature (IR thermometer and IR thermal camera) IR: infrared

In contrast to the IR thermometer readings, thermal imaging conducted using an IR thermographic camera revealed a lower mean facial skin temperature at baseline. In the test arm, the baseline temperature was recorded as 32.22 ± 0.85 °C. Following the exercise intervention, which was intended to elevate peripheral circulation and simulate thermoregulatory demand, a statistically significant decrease in mean temperature to 31.60 ± 0.83 °C was observed (CFB -0.62 °C; p < 0.0001). This reduction may be attributed to evaporative cooling from perspiration, individual variability in thermoregulatory mechanisms, or measurement sensitivity specific to thermographic imaging. Subsequent cleansing with the rinse-off product led to a further pronounced reduction in facial skin temperature to 29.85 ± 1.10 °C (CFB: -2.37 °C; p < 0.0001), indicating a robust immediate cooling effect post-application. In the control arm, the baseline mean temperature was 31.99 ± 0.96 °C. Following exercise, the temperature declined to 31.39 ± 0.98 °C (CFB: -0.60 °C; p < 0.0001), remaining within a similar thermal range to the test arm at this time point. After water-only facial rinsing, the mean temperature further decreased to 31.06 ± 1.10 °C (CFB: -0.93 °C; p < 0.0001). Although a cooling effect was also evident in the control arm, the magnitude of temperature reduction was notably less than that observed with the test product, supporting the enhanced efficacy of the rinse-off formulation in delivering perceptible cooling benefits (Figure 3).

Compared to the IR thermometer, the IR thermal imaging method demonstrated greater sensitivity and resolution in detecting temperature changes, making it a more effective tool for evaluating immediate cooling effects on facial skin.

## Discussion

The present internal method development exercise successfully demonstrated the feasibility of using both IR thermographic imaging and non-contact infrared thermometry to assess dynamic changes in facial skin surface temperature in response to physical exertion and the use of a rinse-off product. The study included a control arm, which enabled comparative evaluation of temperature modulation between the test product and water-only application, thereby enhancing the interpretive value of the findings.

Contrary to the expected physiological response in which physical activity typically causes vasodilation and increases peripheral skin temperature due to elevated blood flow, a slight yet statistically significant reduction in facial skin temperature was observed following exercise in both the test and control arms. This response may be attributed to evaporative heat loss driven by increased respiratory rate and airflow across the facial surface during physical exertion, resulting in localized cooling, a phenomenon well-documented in thermo physiological studies of facial microclimate regulation [5,8].

Following the application of the rinse-off facial cleanser, a further reduction in skin temperature was observed, which was more pronounced in the test arm compared to the control. This drop reached statistical significance, particularly when assessed with IR thermographic imaging, supporting the sensitivity of this modality. Infrared thermography enables non-invasive, high-resolution, two-dimensional thermal mapping of the skin surface, with greater sensitivity to localized temperature changes compared to point-based infrared thermometers [9,10]. The latter, although easy to use, monitors temperature at a single site and may not fully capture spatial variability, especially on heterogeneous skin surfaces [11]. These results strengthen the usefulness of IR imaging in dermatological research, particularly for evaluating rapid thermal responses to cosmetic products [5].

The standardized method developed in this study holds strong potential for adaptation beyond rinse-off facial cleansers. Its applicability can be extended to a variety of topical products such as gels, creams, serums, lotions, and leave-on formulations that claim to provide cooling, soothing, or warming effects. By adjusting the assessment timelines and incorporating additional endpoints, such as subject-reported sensation scores or long-term temperature monitoring, the methodology can be customized to evaluate sustained or delayed thermal responses. Additionally, this approach may be integrated into broader clinical research for dermatological conditions where temperature modulation is relevant, such as post-laser recovery, inflammatory dermatoses, or vascular skin disorders. Its non-invasive, objective, and reproducible nature makes it particularly valuable for evaluating product performance in both healthy and sensitive skin populations.

This internal standardization study, while methodologically sound, is subject to several limitations. First, the evaluation was conducted on a small, homogenous population of healthy male volunteers, which may limit the generalizability of the findings to broader clinical or consumer populations, including women and individuals with underlying skin conditions. Second, although both IR thermography and infrared thermometry were utilized, only immediate post-application effects were assessed; long-term thermal responses were not investigated. Additionally, point-based infrared thermometers lack spatial resolution, potentially limiting their ability to capture temperature variations across different facial regions. The open-label, non-randomized design may have also introduced observer or measurement bias, despite efforts to standardize procedures. Moreover, the influence of confounding variables such as individual differences in sweat rates, baseline skin hydration levels, and room airflow conditions was not fully controlled and may have impacted temperature readings. These limitations highlight the need for larger, randomized controlled trials with broader participant demographics and stricter environmental controls to further validate and refine the utility of this method in real-world clinical and cosmetic settings.

## Conclusions

This internal exploratory evaluation demonstrated the utility of non-invasive infrared thermography and non-contact infrared thermometry in detecting subtle facial skin temperature changes following physical activity and topical product application. In the test arm (rinse-off face wash), both instruments recorded measurable temperature reductions, with IR thermography providing more pronounced and region-specific data. Notably, a slight but significant temperature decline was observed post-exercise, possibly due to localized evaporative cooling. In the control arm (water), temperature changes were minimal and consistently lower than those observed with the test product, highlighting the added cooling effect of the rinse-off product. These findings support the feasibility of standardized thermal assessment methods for future cosmetic product evaluations with cooling or sensorial claims. However, future research is warranted to validate this methodology in larger, more diverse populations, including women and individuals with varying skin types or dermatological conditions, and to assess the long-term thermal effects of topical formulations. Incorporating subject-reported sensory feedback and evaluating product performance under different environmental conditions may further strengthen the robustness and applicability of this approach in real-world settings.
